# Condyle modeling stability, craniofacial asymmetry and ACTN3 genotypes: Contribution to TMD prevalence in a cohort of dentofacial deformities

**DOI:** 10.1371/journal.pone.0236425

**Published:** 2020-07-29

**Authors:** Romain Nicot, Kay Chung, Alexandre R. Vieira, Gwénaël Raoul, Joël Ferri, James J. Sciote

**Affiliations:** 1 Department of Oral and Maxillofacial Surgery, Univ. Lille, Inserm, CHU Lille, U1008—Controlled Drug Delivery Systems and Biomaterials, Lille, France; 2 Department of Orthodontics, Temple University, Philadelphia, PA, United States of America; 3 Department of Oral Biology, University of Pittsburgh School of Dental Medicine, Pittsburgh, PA, United States of America; Ohio State University, UNITED STATES

## Abstract

Craniofacial asymmetry, mandibular condylar modeling and temporomandibular joint disorders are common comorbidities of skeletally disproportionate malocclusions, but etiology of occurrence together is poorly understood. We compared asymmetry, condyle modeling stability and temporomandibular health in a cohort of 128 patients having orthodontics and orthognathic surgery to correct dentofacial deformity malocclusions. We also compared *ACTN3* and *ENPP1* genotypes for association to clinical conditions. Pre-surgical posterior-anterior cephalometric and panometric radiographic analyses; jaw pain and function questionnaire and clinical examination of TMD; and SNP-genotype analysis from saliva samples were compared to assess interrelationships. Almost half had asymmetries in need of surgical correction, which could be subdivided into four distinct morphological patterns. Asymmetric condyle modeling between sides was significantly greater in craniofacial asymmetry, but most commonly had an unanticipated pattern. Often, longer or larger condyles occurred on the shorter mandibular ramus side. Subjects with longer ramus but dimensionally smaller condyles were more likely to have self-reported TMD symptoms (*p* = 0.023) and significantly greater clinical diagnosis of TMD (*p* = 0 .000001), with masticatory myalgia most prominent. Genotyping found two significant genotype associations for *ACTN3* rs1671064 (Q523R missense) *p* = 0.02; rs678397 (intronic SNP) *p* = 0.04 and one significant allele association rs1815739 (R577X nonsense) *p* = 0.00. Skeletal asymmetry, unusual condyle modeling and TMD are common and interrelated components of many dentofacial deformities. Imbalanced musculoskeletal functional adaptations and genetic or epigenetic influences contribute to the etiology, and require further investigation.

## 1. Introduction

Growth and stability of the mandibular condyle is essential for attainment and maintenance of mandibular size and morphology. Agenesis, trauma, local infectious pathologies and juvenile idiopathic arthritis all produce similar and distinctive mandibular morphologic disruptions, due to decreased growth in length and normal attainment of transverse width. [1] These condylar growth deficiencies result in skeletal class II open bite malocclusions characterized by a downward and backward growth rotation at the joint articulation and a pronounced antigonial notch. Variability in diminished mandibular length and severity of the dysmorphology is directly related to the chronologic age at which condylar disturbance is first encountered, as demonstrated in case reports of patients with either infections or trauma. [2] In normal joint growth, the condyles are adaptive to variable forces produced by differences in jaw morphology and muscle function. [3] This can result in quite variable changes in length, area and orientation when jaw growth is imbalanced or disproportionate. [4] When transverse skeletal or dental imbalances develop, the condyles adapt by not obtaining normal growth in size, especially in the medio-lateral dimension. [5] These transverse adaptations are reportedly more at risk for development of condyle displacement within the joint and temporomandibular joint disorders (TMD). [6–8]

Dentofacial deformity patients develop the most disproportionate skeletal variations of normal growth and are most likely to have TMJ dysfunction and symptoms. [9] Orthodontic and orthognathic surgical treatments have recently been documented as effective therapies in restoring facial balance and relieving TMD signs and symptoms, especially for related arthralgia or myalgia. [10,11] TMD is also more likely to be associated with dentofacial deformities when a component of the malocclusion involves a significant imbalance in facial symmetry. [12] Well know arthritic conditions like idiopathic condylar resorption may produce skeletal malocclusions and TMD, but in most dentofacial deformity patients condylar modeling is more subtle, and therefore not always considered in treatment planning and outcomes. We recently developed a method for measuring normal condyle geometry variations in a group of patients with dentofacial deformities which revealed differences in condylar length or area between left and right sides. [13] Through genetic analysis we identified a genetic variant in the *ENPP1* gene (rs937300) which associated with these variations as a potential causal factor, since it functions as an inhibitor of hydroxyapatite formation during mineralization. The finding indicates that some individuals may be more susceptible to condyle modeling due to both functional influences and inherent quality of bone adaptation.

When craniofacial asymmetry was present, these patients reported a significantly elevated level of pain and jaw dysfunction. [14] This coincided with significantly elevated clinical diagnosis of disc displacement with reduction, myalgia, arthralgia and TMD related headache. In discriminating between different patterns of asymmetry, we developed a new posterior anterior cephalometric analysis which distinguishes four anatomic subclassifications (group one—four), each with a different rate of TMD symptoms. The mandibular asymmetry categories are described in the Materials and Methods, Section 2.3. In group three, chin deviation is displaced to the side of the face which also has the longer ramus length. This unusual subclassification of asymmetry is very common and results in the highest rate of patient reported TMD symptoms. [14] Genetic analysis revealed that an additional variant in *ENPP1* (rs858339) associated with this asymmetry pattern. Group two and three had the highest rates of reported TMD symptoms, and four had the lowest—even though skeletal imbalance was the most pronounced in this group. Therefore the posterior anterior cephalometric classification of asymmetry may indicate which groups are at higher risk for having or developing TMD, but does not discern which individuals within a group are predisposed. Although other predisposing factors such as variations in the functional environment are arguably a primary factor influencing TMD, two possible explanations could be differences in condyle geometry variation, during or after growth, and genotype.

Since mandibular morphology is a heritable trait, it is important to consider genetic and epigenetic (functional) influences upon condylar growth and adaptation. [15,16] Fibroblast growth factor 2 (FGF-2) is a primary growth promoter of condylar cartilage growth during development. [17] In animal models where lateral functional shift of the mandible are introduced, condylar FGF-2 expression is increased on the protruded ramus side and decreased on the contralateral retrusive side, introducing asymmetric changes in chondrocyte activity and cartilage morphology. [18] FGF-2 promotes ENPP1 activity, resulting in enhanced subcondral bone mineralization. [19] *ENPP1* has at least 66 functional variants, some of which might respond differently to condylar environmental influences. [20] Therefore, changes in left versus right condyle morphology demonstrated in condyle geometry variation could be the result of developing facial asymmetry during growth, rather than the primary cause. An additional influence on ENPP1 expression is *ACTN3* genotype. In Actn3-/- mice *ENPP1* gene expression is increased, resulting in lowered limb bone mineralization apposition rate, trabecular number and bone volume. [21] We recently associated the common *ACTN3* R577X mutation which results in lack of protein expression, with skeletal Class II malocclusion. The initial hypothesis is the lack of ACTN3 protein results in diminished subcondral bone growth or maintenance through increased ENPP1 activity. [22]

To further understand variation in presentation of TMD signs and symptoms, we evaluated how different patterns of craniofacial asymmetry, asymmetric condyle geometry variation and *ENPP1* or *ACTN3* genotypes might interact, in a cohort of dentofacial deformities subjects already included in previous studies. [13,14,22,23] These findings may be of diagnostic predictive value in counseling patients for their potential risk for developing or aggravation of TMD.

## 2. Materials and methods

### 2.1 Subjects

Subjects with dentofacial deformities who were undergoing elective orthognathic surgery for correction of dento-maxillo-facial dysmorphology (normal variations in jaw geometry which produce malocclusion and facial imbalance) were recruited for study from the Department of Oral and Maxillofacial Surgery, Roger Salengro Hospital, Lille France, after signing an informed consent to participate. The clinic serves an area of northern France of about 4 million inhabitants under the country’s National Health Service, and is the region’s primary center for maxillofacial surgery. The population for recruitment were non-growing adolescents or adults with a mean age of 26 years and 76% female. They were undergoing combined orthodontic and surgical treatments which included pre-surgical orthodontics, at least a mandibular bilateral sagittal split osteotomy, in conjunction with Lefort osteotomies of the midface as necessary, and a second round of post-surgical orthodontics to finalize occlusion. The study included subjects without other systemic conditions, and excluded those undergoing surgery for facial trauma, tumor, condylar hypertrophy or idiopathic resorption, rheumatoid or osteoarthritis, and congenital craniofacial syndromes or developmental conditions that might influence craniofacial growth. [24] Clinical diagnoses of each patient were summarized at the time of surgery to include the sagittal and vertical malocclusion classification, based upon the extent of required sagittal, vertical and transverse repositioning of jaws estimated in the surgical treatment plan. De-identified information for study included radiographic and diagnostic images, calibrated for magnification, details of the surgery along with information for height, weight, race, ethnicity, age and sex. Subjects signed an informed consent form, and the research protocol was validated by the French independent ethical committee (Certificate CPP12/44), the Temple University Temple (Certificate 13438) and the University of Pittsburgh institutional review boards (Certificate PRO12080373).

### 2.2 Condyle geometry variation assessment

**Fig 1:**
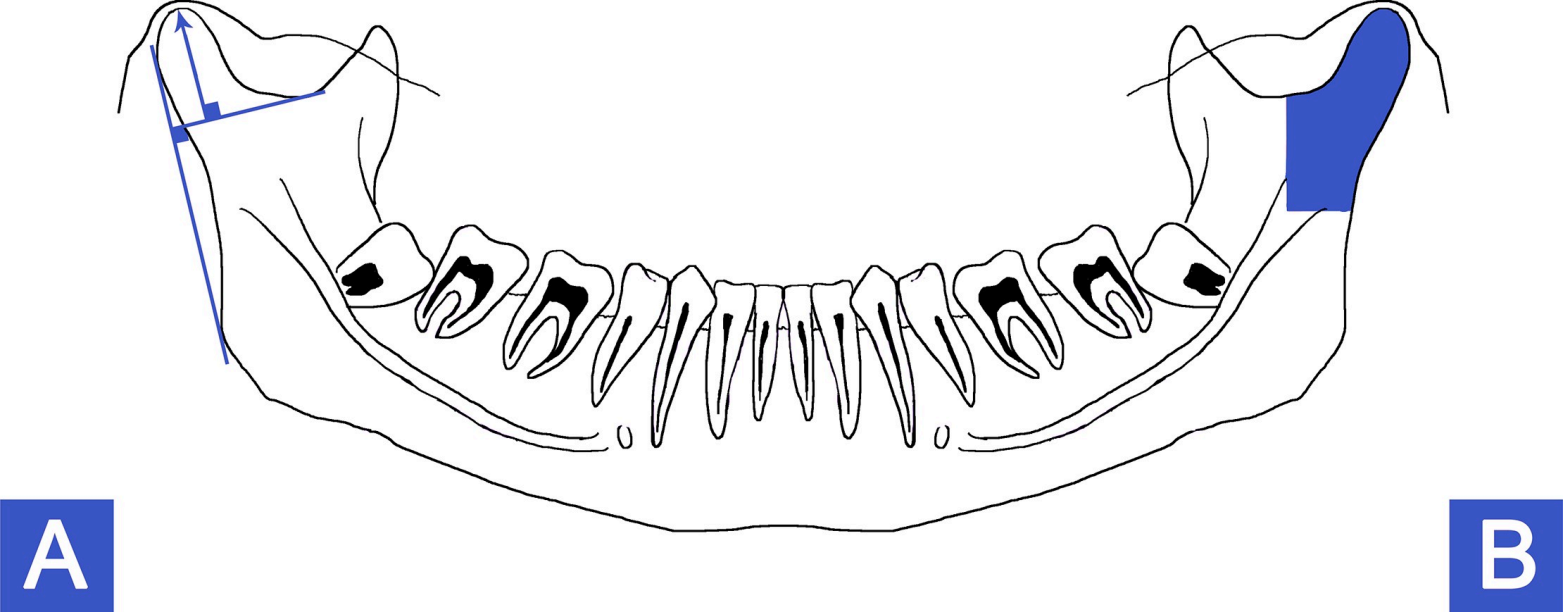
Panoramic landmarks related to condyle modelling measurements. A–Two lines were constructed to evaluate condylar height, one drawn tangential to the posterior edge of the mandible passing through the most posterior points of the condyle and mandibular ramus, and the perpendicular line passing through the lower end of the mandibular notch. Condylar height was measured perpendicular to the latter between the mandibular notch and the highest point of the condylar unit. B—The surface of the condylar unit was measured, contouring the lowest point of the mandibular notch to the *lingula mandibulae*, then perpendicular to the rear edge of the mandibular ramus. Bone modeling was determined by a differential measurement of condylar height or condylar surface defined by a percentage in relation to the larger side between right and left sides on a pre-surgical panoramic radiograph.

Although there is no widely accepted method to assess condyle modeling as part of normal growth or physiologic adaptation after maturation, the metric method (two dimensional radiographic measurements) have historically been utilized. [25,26] In our patient population, we recently developed a metric measurement method that compares morphometric differences between left and right condyle height or condyle area on panoramic radiographs. [13] Two lines were constructed to evaluate condylar height, one drawn tangential to the posterior edge of the mandible passing through the most posterior points of the condyle and mandibular ramus, and the perpendicular line passing through the lower end of the mandibular notch. Condylar height was measured perpendicular to the latter between the mandibular notch and the highest point of the condylar unit (Fig 1A). The surface of the condylar unit was measured, contouring the lowest point of the mandibular notch to the *lingula mandibulae*, then perpendicular to the rear edge of the mandibular ramus (Fig 1B). Bone modeling was determined by a differential measurement of condylar height or condylar surface defined by a percentage in relation to the larger side between right and left sides on a pre-surgical panoramic radiograph. From this patient data we were able to associate a genetic variant in *ENPP1* with mandibular condyle geometry variation. [13] For comparison, we utilized this existing data base, in combination with an assessment of craniofacial asymmetry to determine associations with TMD or genetic variations. Differences greater than 3% between sides were considered positive for condyle modeling and recorded as percentage difference between sides. Differences less than 3% were recorded as no difference between sides or 0%. Landmarks have been defined on calibrated radiographs, using a cephalostat. Data acquisition has been performed by two observers, jointly defining the landmarks. All measures were done using ImageJ software (National Institute of Health, Bethesda, MD, USA).

### 2.3 Asymmetry assessment and classification

**Fig 2:**
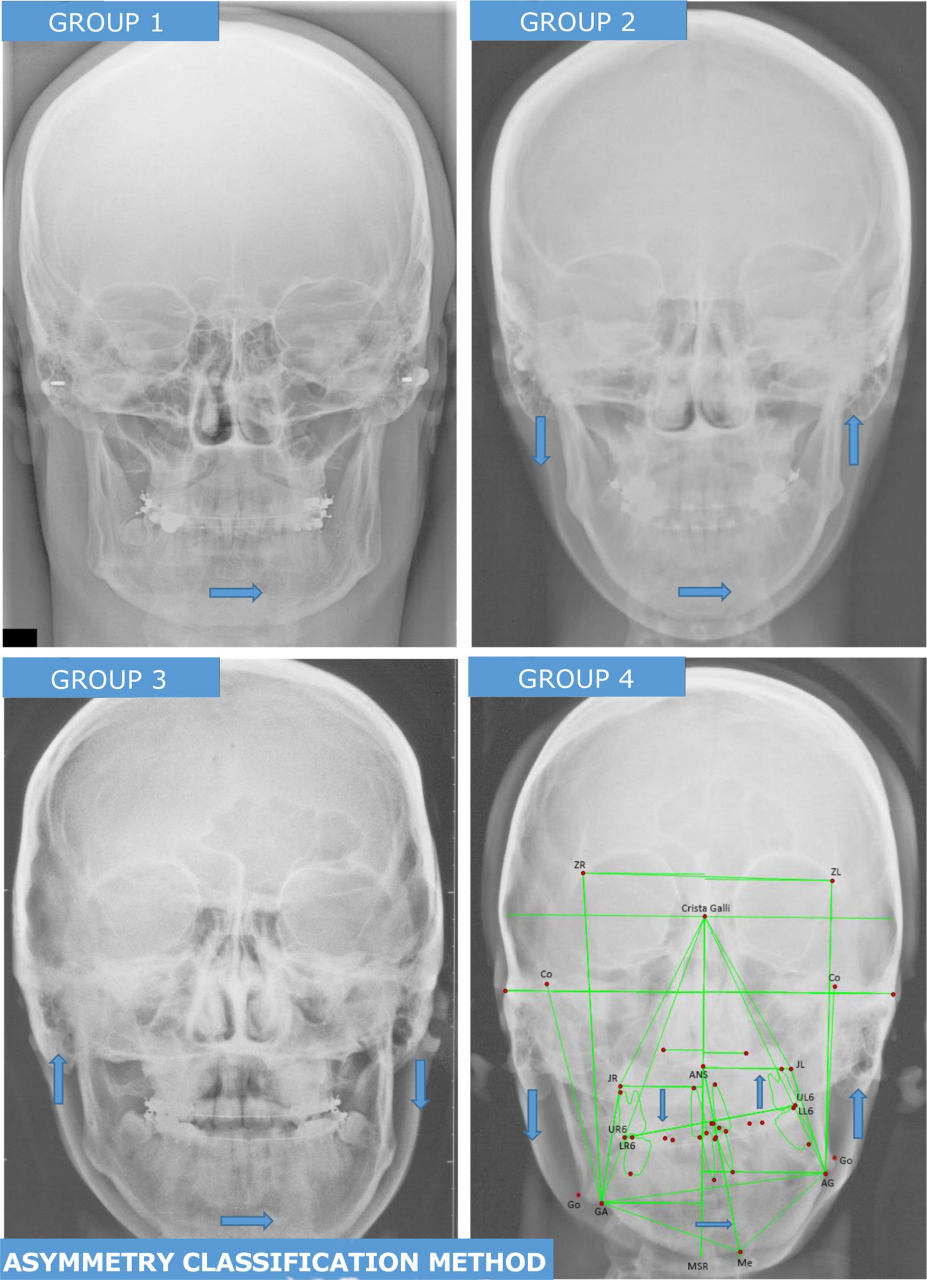
Prototypes for four asymmetry subtypes and illustration of PA cephalometric tracing. Group 1—mandibular body asymmetry, but symmetry in mandibular rami (sometimes termed “mandibular yaw”); Group 2—ramus asymmetry: differences in left and right ramus heights with mandibular chin deviated towards the shorter ramus height side; Group 3—atypical asymmetry: differences in ramus heights with mandibular chin point deviated towards the longer ramus height side; and Group 4—C-shaped asymmetry: differences in left and right ramus heights, with mandibular chin deviated towards the shorter ramus height side (as with group 2) but in addition with pronounced maxillary midfacial canting. Landmarks used for cephalometric analysis labeled on Group 4.

Craniofacial asymmetry is a type of dentofacial deformity which has a unique set of morphologic variations for which there have been many classification approaches. We recently developed a new diagnostic assessment based upon 17 anatomic landmarks on posteroanterior cephalometric radiographs. [14] These landmarks were converted into 6 cephalometric metric assessments which characterized four different asymmetry subtypes present in the population (Fig 2). We characterized these as group 1: asymmetry of the mandibular body, but symmetry in mandibular rami (sometimes termed “mandibular yaw”); group 2: differences in left and right ramus heights, with mandibular chin deviated towards the shorter ramus height side (what most clinicians would refer to as a typical facial asymmetry); group 3: differences in ramus heights with mandibular chin point deviated towards the longer ramus height side (an “atypical” facial asymmetry); and group 4; differences in left and right ramus heights, with mandibular chin deviated towards the shorter ramus height side (as with group 2) but in addition with pronounced maxillary midfacial canting. From the cephalometric analysis patients were classified as symmetric or asymmetric, and if asymmetric into subtypes. From these patient groupings we previously found asymmetry group 2 and 3 had the highest incidence of pre-surgical TMD, and groups 1, 3 and 4 had significant associations with genetic variants in *ENPP1* [14]. In the present study we compared these classifications for asymmetry to differences on condyle geometry, as determined in section 2.2.

### 2.4 Assessment of temporomandibular disorders

Temporomandibular joint functioning was assessed as a routine part of the pre-surgical evaluation using the Diagnostic Criteria for TMD (DC/TMD). [27] Overall this young population is not presenting with fibromyalgia or pain related disability diagnosed in Axis II of the diagnostic criteria. The three common Axis I disorders associated with asymmetry in the population were disc displacement with reduction (DDR) (78%), myalgia (61%) and arthralgia (33%). [14] We use the jaw pain and function (JPF) questionnaire to assess patient reported symptoms as an indication of perceived severity before and one year after jaw surgery. [23] The JPF was developed as a simple screening tool to determine presence of TMD. [28] It consists of eight questions about jaw pain and five questions related to jaw function. The questionnaire has been validated to reliably distinguish between normal (scores < 6) and TMD subjects (scores ≥ 6) with up to 98% sensitivity and 100% specificity. [29] It has been validated in European translations [30] and we use a French version. [14,23] In this assessment, we included TMD patients with positive diagnosis for DDR, myalgia and/or arthralgia. Patients with positive clinical diagnosis for other, less common forms of TMD were excluded from study since they were insufficient number to investigate.

### 2.5 Comparing condyle variation with facial asymmetry

A total of 128 subjects had complete data sets for comparison of condyle variation with symmetry classification. We compared condyle height or condyle surface as percent differences between sides, and which side, either left or right, was longer or larger to the symmetry classification of patients. Symmetric subjects and those in asymmetry group 1 had equal left and right mandibular ramus length. In the other three asymmetry groups one ramus was larger in length and one smaller. In groups 2 and 4 the chin, as indicated by the mandibular menton landmark, was deviated away from the facial midline towards the shorter ramus length side. In group 3 the chin however was deviated away from the facial midline towards the longer ramus length side.

In comparing condyle differences to these patterns of chin and ramus asymmetry, we anticipated finding that the longer or larger condyle would be located on the same side as the longer ramus. However, this was not true in the majority of patients. Rather, it was more likely that increased condyle dimension was located on the side with the shorter ramus dimension. Because of this unexpected finding, we further classified asymmetry groups into those who followed the normal, expected pattern or those with an unexpected pattern as follows: (Table 1).

**Table 1:** Criteria for normal vs. abnormal condyle variation in asymmetry condyle height or area difference.

same side as menton deviation	opposite side as menton deviation
Group 1—abnormal pattern	Group 1—normal pattern
Group 2—abnormal	Group 2—normal
Group 3—normal	Group 3—abnormal
Group 4—abnormal	Group 4—normal

**Fig 3:**
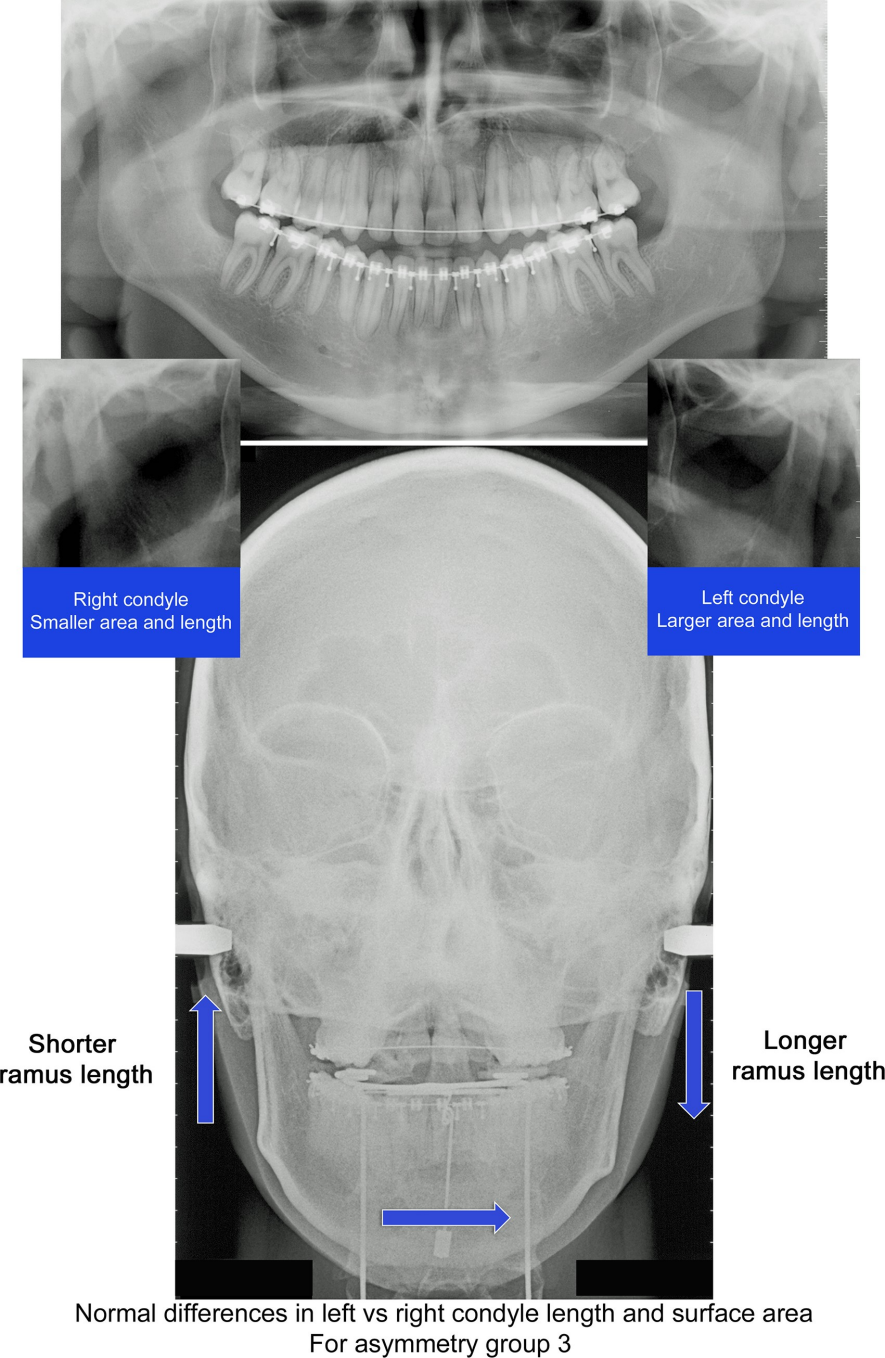
Illustration of normal condyle modeling. Subject from asymmetry group 3, atypical asymmetry, with menton deviated toward longer ramus side. Condyle geometry variation defined as “normal” due to longer and larger condyle on side with longer ramus length.

**Fig 4:**
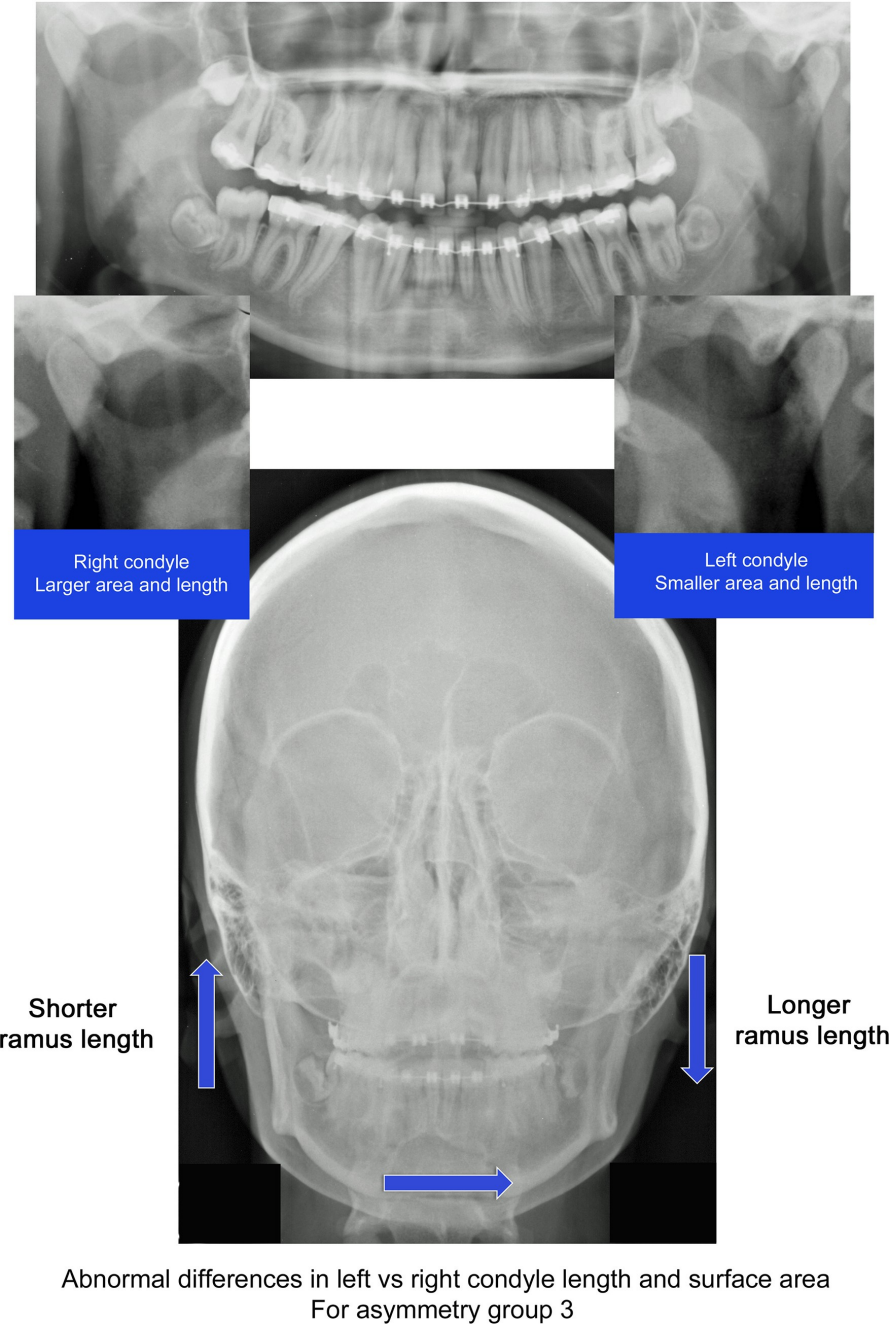
Illustration of abnormal condyle modeling. Subject from asymmetry group 3, atypical asymmetry, with menton deviated toward longer ramus side. Condyle geometry variation defined as “abnormal” due to longer and larger condyle on side with shorter ramus length.

This criteria recognizes that in asymmetry group 3 the menton is deviated towards the shorter ramus side, but since the ramus is longer on this side, it is anticipated that condyle dimension would also be larger. Figs 3 and 4 compare of one subject in group 3 which had normal condyle modeling (1) and one with abnormal modeling (2).

Based upon this realization the anatomical investigation to study had two primary endpoints: 1) determine the frequency of condyle variation in patients with symmetry compared to asymmetry and 2) determine if condyle variation could have contributed to differences in TMD, in the differing patterns of asymmetry.

### 2.6 Genotyping

Saliva samples were collected during the pre-surgical evaluation and processed utilizing DNA Genotek kits. Genomic DNA was used for profiling of polymorphisms using TaqMan chemistry [31] and for sequencing using an automatic sequence-detection instrument (ABI Prism 7900HT, Applied Biosystems). Seven single nucleotide polymorphisms were selected if genotyping: in *ACTN3* rs1671064, rs1815739 and rs678397 [22] and in *ENPP1* rs937300, rs6569759, rs858339, and rs1409181. [23] The asymmetry population was compared for SNP variants between normal versus abnormal modeling, as summarized in Table 1, section 2.5.

### 2.7 Statistical testing

Differences in condyle height or condyle area were compared between all symmetric and all asymmetric subjects using an unpaired *t* test, and an ANOVA for comparison between the different asymmetry groups. For relationship to TMD, JPF scores were compared for each individual asymmetry group between normal growth and abnormal growth by individual unpaired *t* tests. In cases where individual asymmetry group comparisons revealed no significant differences for JPF, all asymmetry groups were averaged together (normal vs. abnormal condyle height and area), and compared by Student *t* tests to determine significance. For clinical diagnoses of TMD, Chi-square tests were used to compare individual and all TMD diagnoses between individual asymmetry groups, and for the number of TMD diagnosis between all normal vs. abnormal condyle height and area groups. Chi-square and Fisher’s exact tests were used to determine the over-representation of genotypes and alleles.

## 3. Results

### 3.1 Differences in condyle modeling between symmetry and asymmetry

Complete data was available from 128 subjects, 56 were classified within one of four craniofacial asymmetry groups from posterior anterior cephalometric analysis. When compared for differential bone modeling of mandibular condyles from panoramic radiographic analysis, there were very significant differences between symmetric vs asymmetric subjects. In the symmetric group there was a mean condyle height variation between sides of 7.37% ± 5.49, compared to 10.89% ± 7.39 for the asymmetric group, which was significantly different (*p* = 0.0025). The condyle area mean difference in symmetrics was 8.22% ± 5.53, while the asymmetric group difference was 10.35% ± 8.35, which was nearly significantly different at (*p* = 0.08).

Within the asymmetry population individual groups were compared to determine if there was a difference in the amount of left compared to right condyle height or area modeling. There was no significant differences for condyle height, but there was a significant difference for condyle area (*p* = 0.02). For condyle area, the amount of difference between sides increased as the severity of asymmetry became more pronounced. Group 1 had a mean area difference of 4.41%, group 2–11.62%, group 3–14.17% and group 4–16.56%.

### 3.2 TMD differences in asymmetries between normal and abnormal condyle modeling

We investigated whether normal vs abnormal condyle growth modeling, based upon study criteria (Table 1), might contribute to differences in TMD prevalence. This revealed that abnormal condyle modeling was the most common finding throughout the classifications of asymmetry (Table 2). For condylar height, abnormal modeling ranged from 50 to 70 percent. For condylar area, modeling rates were higher in most groups, at rates between 50 and 75. Only asymmetry group 2 had and almost equal distribution of normal versus abnormal modeling.

**Table 2:** Condyle modeling proportions in asymmetries.

	height modeling				area modeling			
	abnormal		normal		abnormal		normal	
	n	%	n	%	n	%	n	%
asymmetry group 1	5	62.5	3	37.5	6	75	2	25
asymmetry group 2	11	48	12	52	12	52	11	48
asymmetry group 3	12	66.6	6	33.3	13	72	5	28
asymmetry group 4	5	71	2	29	4	57	3	43

We compared patient reported TMD symptoms using the JPF questionnaire between normal and abnormal condyle height or area, for each asymmetry group. The abnormal condyle height group had a mean JPF score or 6.3 while the normal group score was 4.7, demonstrating an almost significant difference in symptoms *p* = 0.055. Those with abnormal condyle area reported mean JPF score of 5.8 compared to the normal area group score of 5.5, resulting in no significant difference *p* = 0.75. When abnormal condyle height or area were grouped together for the entire population, there was an elevation in mean JPF score to 6.65 by comparison to 5.33 in normal modeling group (*p* = 0.023). Patient symptoms were greater in asymmetry groups 2 and 3, and either abnormal height or areas contributed to pain and functional differences at approximately the same rate.

Clinical diagnosis of TMD was compared between normal and abnormal modeling for the population. Since multiple positive diagnoses were common, we first assessed if TMD was present or absent, regardless of single or multiple diagnoses in each patient, which we called “all TMD” patients. This overall positive versus negative comparison revealed a strong trend with a relative higher prevalence of ‘all TMD” in the abnormal group (*p* = 0.05) (Table 3). In individual TMD diagnoses of headache, myalgia, arthralgia, disc displacement with reduction and disc displacement without reduction there were no significant differences. However, there was a trend for diagnosis of myalgia (*p* = 0.06). When total, multiple individual diagnoses were grouped for overall comparison, there was affirming data that abnormal condyle modeling resulted in increased problems with TMD (*p* = 0 .000001).

**Table 3:** Differences in TMD diagnosis between normal and abnormal condyle modeling.

Condyle	group 1		group 2		group 3		group 4		total		
area + height	abnormal (11)*	normal (5)	abnormal (23)	normal (23)	abnormal (25)	normal (11)	abnormal (9)	normal (4)	abnormal (68)	normal (43)	Chi-squared test
all TMD	3	1	13	8	11	3	1	0	28	10	*p* = 0.05
headache	0	0	3	1	2	0	1	1	6	2	*p* = 0.41
myalgia	3	1	8	5	15	2	1	2	27	10	*p* = 0.06
arthralgia	0	0	3	4	6	4	0	0	9	8	*p* = 0.42
DDR	2	0	9	5	15	6	1	1	27	12	*p* = 0.19
DD w/o R	0	0	2	0	5	1	0	0	7	1	*p* = 0.11
total positive TMD Diagnoses (including multiple diagnosis for each subject)									68	33	*p* = 0 .000001

*(n) indicates total number of subjects per group; DDR = Disc displacement with reduction; DD w/o R = Disc displacement without reduction without limited opening.

### 3.3 Genotype differences

For genotype comparisons we grouped all abnormal condyle height and abnormal condyle area modeling subjects together and compared them to those with normal modeling in each individual asymmetry group. For *ENPP1* there were no significant differences in genotypes or alleles for SNPs rs937300 (*p* range values 0.29 to 0.88), rs6569759 (p range values 0.85 to 0.92) or rs858339 (p range values 0.37 to 0.51). For *ACTN3* however there were significant differences in group 2 for genotypes rs1671064 (*p* = 0.02), rs678397 (*p* = 0.04) and alleles 1815739 (*p* = 0.00) (Table 4). There was a trend for rs1815739 genotypes (*p* = 0.08). Results were most likely positive in group 2 since it had the most subjects for comparison.

**Table 4:** Comparison of SNP genotypes by condyle modeling pattern.

Gene	ACTN3	rs1671064 (Q523R)			p value	rs1815739 (R577X)			p value	rs678397 (intronic)			p value
SNP		GG	GA	AA	genotype/allele	TT	CC	TC	genotype/allele	TT	CC	CT	genotype/allele
Group 1	normal	2 (15)	7 (54)	4 (31)	*p* = 0.15/ 0.08	2 (15)	4 (31)	7 (54)	p = 0.11/ 0.07	2 (15)	4 (31)	7 (54)	*p* = 0.15/ 0.07
	abnormal	2 (66)	1 (33)	0		2 (66)	0	1 (33)		2 (66)	0	1 (33)	
Group 2	normal	5 (22)	18 (78)	0	*p* = **0.02**/ 0.08	9 (33)	1 (4)	17 (63)	p = 0.08/ **0.00**	10 (40)	1 (4)	14 (56)	*p* = **0.04**/ 0.08
	abnormal	5 (26)	9 (48)	5 (26)		5 (26)	6 (32)	8 (42)		6 (31)	6 (31)	7 (38)	
Group 3	normal	2 (8)	14 (61)	7 (31)	*p* = 0.69/ 0.49	2 (4)	6 (33)	16 (63)	p = 0.84/ 0.34	2 (10)	7 (33)	12 (57)	*p* = 0.72/ 0.64
	abnormal	2 (12)	11 (69)	3 (19)		2 (12)	3 (19)	11 (69)		2 (15)	3 (21)	9 (64)	
Group 4	normal	4 (40)	6 (60)	0	*p* = 0.73/ 0.79	4 (40)	0	6 (60)	p = 0.3/ 0.79	4 (40)	2 (20)	4 (40)	*p* = 0.62/ 0.45
	abnormal	2 (50)	2 (50)	0		2 (50)	0	2 (50)		2 (50)	0	2 (50)	

## 4. Discussion

### 4.1 Condylar role in mandibular modeling

Modeling is the process by which bone enlarges and takes shape during normal growth. Modeling is a complimentary process of resorption or deposition to generate new tissue during homeostasis or for modification of size and shape. The condyle contributes to both mandibular modeling and modeling during normal growth, and has inherent capacity to remodel after growth is completed through chondrocyte sensitivity to variations in mechanotransduction. [32] These effects are well documented in orthodontic treatment of Class II malocclusions with repositioning appliances, where condyles enlarge in anterior-posterior dimension, compared to untreated controls in adolescent or even young adult patients. Ramus modeling can also occur at the same time as changes in condyle dimensions. [33] This ability to adapt and transition from chondrogenesis to osteogenesis is a unique anabolic feature of condylar cartilage. In environments where forces differ in the transverse occlusal plane due to crossbite, some reports have identified asymmetrical condylar modeling, while other have not. In most studies considering skeletal asymmetries of the mandible, condyles are reported to be asymmetric, with decreased cross sectional area, surface size and ramal height on the deviated side. [34,35]

As descriptive morphology of craniofacial asymmetries has advanced, several classification approaches which emphasize mandibular roll, pitch and yaw have established that ramus length, menton deviation and condylar morphological variations do not always match each other, and ramus height may be longer on the same facial side to menton deviation. [14] We recently developed a posterior–anterior celphalometric analysis utilizing six measurements, with four of these detecting mandibular differences between the body, width, ramus length and menton deviation. When viewed by principal component analysis, symmetric and asymmetric faces cluster as distinct groups. Variability in asymmetric groups 1–4 revealed that principal components clustered by differences between the left and right mandibular sides, indicating that a consistent geometric variability explained differences in morphology between them. [14] The main fluctuating variable between groups is the relationship of chin deviation in the transverse plane to left vs right ramus length differences between sides. With regard to these relationships, this study has demonstrated two distinct patterns of asymmetry which have not been commonly recognized previously. First, it is almost equally common to have longer ramus length on the same side as chin deviation as it is to have a longer ramus on the contralateral side. Second, the condyle on the longer ramus side may be geometrically smaller than on the shorter ramus side, regardless of the specific menton to ramus relationship. Insight into these different patterns of asymmetry most likely arise from the number of subjects we have been able to evaluate and compare, rather than previous studies with a more limited population from which these more subtle variations would be less possible to distinguish. These findings raise important clinical questions in patient diagnosis, treatment and management, since differences may relate to intrinsic genetic factors and risk of pre or post treatment TMD and stability.

### 4.2 Condyle modeling and signs or symptoms of TMD

In our population, asymmetric subjects have both higher clinical diagnoses of TMD and higher reported TMD symptoms, as indicated by the JPF survey. [14] Yet within individual asymmetric groups, the standard deviation for mean values of patient reported symptoms are quite high. This is especially true for group three which had a mean JPF score of 9.11 ^±^ 5.62. Likewise group two, also with elevated symptoms had JPF score of 6.94 ^±^ 5.46. Therefore we evaluated if the pattern of condyle modeling might influence symptom variability by comparing expected versus unexpected geometry variations (Table 1). This resulted in a significantly elevated (*p* = 0.02) level of patient reported symptoms when modeling did not match with ramus height or area differences. For TMD diagnosis the same comparison revealed a significant difference for subjects with at least one positive finding (*p* = 0.05) (Table 3). While the individual types of TMD were not significant, when the total number of patient diagnoses were compared, there was very significantly elevated differences (*p* = 0.0001). This resulted since those with abnormal condyle modeling most often had multiple combinations of different types of TMD. The most common TMD diagnoses in both normal and abnormal groups were masticatory muscle myalgia and disc displacement with reduction, with abnormals almost three times more likely to have myalgia and twice as likely to have disc displacement. Arthralgia rates were almost equal between groups. In abnormal modeling arthralgia was not nearly as likely, with a ratio of 3:1 compared to myalgia. Since presence of arthritic conditions or condylar resorption were part of the study exclusion criteria, the study considered if differences in condylar modeling produced different rates of arthralgia, and found no difference.

An opposite finding occurred for myalgia and disc displacement with reduction, which are much more likely with abnormal condyle modeling. Myalgia was the only individual TMD diagnosis which was almost significantly different for condyle modeling (*p* = 0.06), and emerged as the solitary clinical diagnosis most related to abnormal modeling (Table 3). The asymmetric patients under study had significant skeletal imbalances, when evaluated by posterior-anterior cephalometric analysis, with the greatest skeletal variation being ramal height differences at p < 0.0001. [14] Muscle functioning is also imbalanced in craniofacial asymmetry, with a significant increase in fast twitch skeletal muscle fibers on the side to which the menton was deviated, when analyzed from masseter muscle biopsy during surgery. [14] Imbalanced force during whole muscle contraction in repetitive athletic activity is a well-known cause of myalgia, especially in the lower back and shoulder. Women athletes with hip strength asymmetry, a type of hip muscle imbalance, are more likely to develop occurrences of low back pain. [36] For male wheelchair athletes, weakness in humeral head depressors, a shoulder muscle imbalance, can result in development of rotator cuff impingement syndrome. Muscle rather than joint pain may also arise from repetitive, unvaried, continuous locomotion and often affect women more than men in the upper extremities. [37]

The TMJ is a unique craniofacial joint since there are three articulations, each joint and the occlusion. Postural and functional position of the jaws is coordinated by afferent input from muscle spindles throughout the head and neck and imbalances in the trigeminal motor system can produce imbalanced stress distribution throughout the cervical spine. [38] Facial skeletal asymmetry produces functional imbalances in masticatory muscles with greater activation on the longer ramus side. This produces an uneven stress distribution in the mandible, which may occur due to either differences in masticatory muscle forces or skeletal geometry. [38] The TMJ can buffer imbalanced mechanical stress by alteration in rates of chondrogenesis, which may be the etiology of abnormal condyle modeling. In animal models, imbalance in masticatory muscle activity results in asymmetric growth of subcondral bone to normalize stress distributions. [39] This presents the interesting possibility that individual patients adapt better to craniofacial asymmetry if condyle geometry differences provide positive stress support within the joint. Imbalances may also explain why patients experience high rates of myalgia, since increased peripheral activation of masseter muscle spindles can contribute to and help maintain chronic muscle pain. [40]

### 4.3 *ACTN3* anpd *ENPP1* genotypes

ENPP1 is a trans membrane glycoprotein which synthesizes inorganic phosphate from extracellular ATP, inhibiting hydroxyapatite formation. SNPs in *ENPP1* are associated with a large number of bone diseases and abnormal bone and joint morphology. Different mechanical strain environments change *ENPP1* expression which can lead to either protection or calcification of endplate cartilage chondrocytes. [41] In the mandible we recently found the rs937300 SNP associated with variation in condyle geometry between left and right sides. [13] The GG genotype was protective against condyle height reduction. Therefore it is likely that both genotype and functional variations contribute to the pattern of condyle modeling. Two SNPs also associated with either group 1 or group 3 craniofacial asymmetry. [14] Therefore, we evaluated the possibility that *ENPP1* variants also contributed to abnormal condyle modeling, but have so far found no associations.

ENPP1 and ACTN3 are connected in bone adaptation by an unknown biologic mechanism. Nevertheless, in Actn3-/- mice osteoblasts have up-regulated expression of ENPP1 which may lead to differences in mineralization rates. [21] This *in vitro* connection is consistent with disruption of normal mineralization resulting in an overall decrease in bone mass in α-actinin-3 deficiency. In humans the *ACTN3* R577X (rs1815739) null polymorphism associates with higher serum levels of modeling markers, which may make bone more susceptible to geometry variations. [42] We found a very significant association (*p* < 0.0001) for R577X allele differences, with the X allele (null polymorphism) elevated in the normal condyle modeling group. There were additional significant associations for *ACTN3* rs1671064 and rs678397 genotypes. rs1671064 is the Q523R polymorphism which produces an A to G transition not know to have functional consequences. Q523R however has been found to have linkage disequilibrium with R577X, [43] and this may indicate that R577X being tested is in close proximity (in linkage disequilibrium) with Q523R, which we could speculate is the actual genetic variant (mutation) that is leading to condyle modeling and TMD symptoms. rs678397 is an intronic SNP which has previously been identified as having a very significant association (*p* = 0.003) with skeletal class II malocclusions, most likely through variations in condylar growth. [22] All of these findings indicate that both *ENPP1* and *ACTN3* genotypes associate with varying patterns of condyle modeling in ways which are not yet understood. *ACTN3* genotypes can influence *ENPP1* expression, as can changes in cartilage mechanical strain environments. [40] Differing biomechanical forces as epigenetic factors and intrinsic genetic differences, both contribute to the pattern of condyle modeling stability or instability, and require further investigation.

### 4.4 Study shortcomings

The study used conventional posterior anterior cephalograms and panoramic radiographs to determine morphologic differences in the pattern of craniofacial asymmetry and condyle modeling. These imaging modalities are routinely utilized in radiographic evaluation of dental patients. Although commuted tomography (CBCT) is more precise, current clinical guidelines from the American Dental Association and American Association and Pediatric Dentistry recommend prescription of panoramic radiographs for routine periodic imaging.

Although the study investigated a relatively large number of patients, the study protocol separated participants almost in half for symmetry, and the asymmetric subjects were eventually sub divided into 8 groups for asymmetry type and condyle remolding differences. This resulted in statistical comparisons between limited numbers of patients between groups, and the most important study shortcoming. Future directions will include ongoing studies with larger subject numbers to further understand how condyle modeling and craniofacial asymmetry arise and interact.

### 4.5 Conclusions

In dentofacial deformity subjects, craniofacial asymmetry, abnormal patterns of condyle modeling and TMD are common comorbidities. Condyle geometry variations between mandibular sides and TMD are more common if asymmetry is present as part of the deformity. Often, asymmetric condyle geometry variation does not match differences in ramus length, found in different classifications of facial asymmetry. TMD signs and symptoms are more likely when condyle variations and ramus asymmetry do not match. The most common TMD diagnosis is masticatory muscle myalgia, which likely results from unequal force distributions. *ACTN3* genotypes under study associate with asymmetric condyle modeling and Q523R (missense) may be in linkage disequilibrium with R577X, the common null polymorphism.

These findings further diagnostic precision for interpreting which individual patients might have or develop TMD with or without symptoms. Those in asymmetry groups two and three with imbalanced condyle geometry variation seem to be most at risk, and this is useful diagnostic information. As patients develop asymmetries during maturation, evaluating these features could be important considerations in patient counseling regarding risks for not undergoing surgical treatment to correct skeletal discrepancies.

## Supporting information

S1 Data (XLS)
